# Conference Report: WORKSHOP ON STANDARDIZA TION NEEDS IN BIOTECHNOLOGY Gaithersburg, MD April 26–27, 1993

**DOI:** 10.6028/jres.099.010

**Published:** 1994

**Authors:** Kenneth D. Cole, Lura J. Powell, G. Larry Eitel

**Affiliations:** Biotechnology Division, National Institute of Standards and Technology, Gaithersburg, MD 20899-0001; Chair, Committee E-48 on Biotechnology, American Society for Testing and Materials, Wolder Engineers & Constructors, Golden, CO 80401

## 1. Introduction

**Fig. 1:**
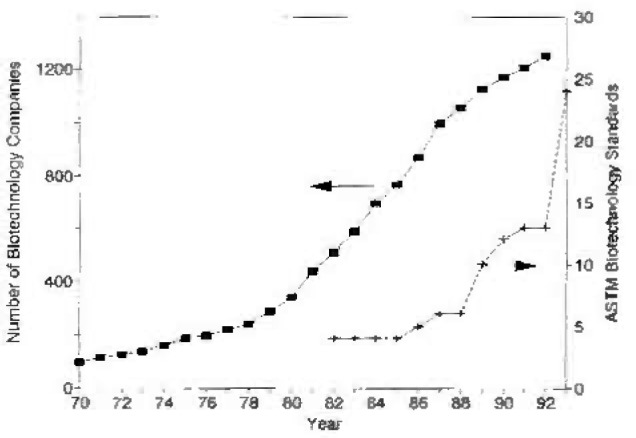
Growth of U.S. biotechnology companies compared to the number of ASTM standards.

The National Institute of Standards and Technology, an agency of the U.S. Department of Commerce, Technology Administration in cooperation with the American Society for Testing and Materials (ASTM) Committee E-48 on Biotechnology held a workshop to determine the necessary standards to aid the rapidly growing biotechnology industry. As shown in Fig. 1, the number of U.S. biotechnology companies grew rapidly from about 100 in 1970 to over 1300 in 1992. The availability of standard methods for the industry fell behind this development curve. Most major biotechnology firms prepared their own procedures for testing in a fragmented approach to using standard procedures. This has handicapped some industry users and complicated the regulatory efforts to validate and qualify new drugs and products that use recombinant organisms in their manufacturing processes.

The ASTM Committee E-48 on Biotechnology was chartered in 1985 to develop test methods, specifications, classifications, practices, guides and terminology standards for the developing biotechnology industry. Where processes use living organisms, the E-48 scope of activity extends from identification and preservation of biological materials, unit processes and their control, enviromental issues, and biomass conversion to validation of biological manufacturing processes.

ASTM Committee E-48 is a dynamic group of more than 100 volunteer scientists, engineers, manufacturers, researchers, government representatives and members of the private sector who develop voluntary consensus-based standards to promote appropriate technological development. The seven technical subcommittees of Committee E-48 and their topics are:

- Materials for Biotechnology: purity and contamination in biological drug products, characterization of proteins, quality control of culture media, synthetic DNA, and preservation methods for biological materials.
- Characterization and Identification of Biological Systems: test methods for plasmids, cell cultures, viruses, bacteria, fungi, and tissues.
- Unit Processes and Their Control: test methods and practices for aseptic sampling, membrane separation, bioleaching, biosensing, and manufacturing equipment performance.
- Environmental Issues: procedures to decontaminate biological process fluids and equipment, containment, safety equipment to avoid biomaterial losses.
- Biomass Conversion: test methods for wood materials, practices for evaluating fuel alcohol plant design and performance, practices for anaerobic/aerobic digesters.
- Biotechnology Equipment Qualification and Process Validation: test methods and practices for validating manufacturing processes and equipment, current good manufacturing practice guidelines for biological processes.
- Terminology: establishing standard terminology for biotechnology.

Over 30 standards have been developed and over 100 more are targeted for development during the next few years by qualified and experienced personnel in this field.

The Workshop on Standardization Needs in Biotechnology was designed to: (1) update industry participants on NIST and ASTM activities that will impact our industry and facilitate regulatory compliance, (2) identify and prioritize needs for consensus, voluntary standards and programs to develop technology, (3) organize resources to develop these voluntary standards, and (4) serve as a forum for industry, universities and government to discuss standards of mutual interest.

The workshop was opened by remarks by Larry Eitel, Chair of ASTM E-48 and Lura Powell, first Vice-Chair of ASTM E-48. The President of ASTM, James A. Thomas gave an overview of the ASTM consensus standardization process. Overviews of the NIST Biotechnology Program, the Standard Reference Materials Program and the Advanced Technology Program were presented. A panel discussion made up of representatives of government agencies discussed federal agency standard issues.

The workshop was then organized into seven workgroups that included: validation and current good manufacturing practices, the environment, characterization and preservation of living cells, safety and transport of biomaterials, bioprocess separations, agricultural/biomass, and microbiological diagnostics. Each workgroup considered the following aspects: a review of current standards, a review of areas where standards might be considered, identification of those areas where standards are needed, prioritization of the standards needs and identification of resources for developing and reviewing standards. The findings of the individual workgroups are presented in this report.

## 2. Validation and Current Good Manufacturing Practices

The validation and current good manufacturing practices workgroup was chaired by Victor Batista, Regional Validation Project Manager for Raytheon Engineers and Constructors. The reference documents (monographs, points-to-consider, guidelines, etc.) already written by professional organizations on validation were identified as a major issue. It was agreed that there is a need to organize and compile this information into a uniform source. The group came to a consensus that the standards should be documents that will be of practical use and have regulatory and scientific depth. Consensus definitions were developed for the three components of a qualification study. The first component is the installation qualification program that will evaluate the documentation (or references) against the system as installed, The second component is the operational qualification program that will provide testing of the system against design specifications and process requirements. The final component is the performance qualification that will provide a scientific study to demonstrate that a process or product will consistently meet the predetermined specifications.

The workgroup decided that standards should contain the following components. An overview is necessary to indicate the purpose of the validation study as well as the data generated and how the information will be used. Installation requirements should contain a clear definition of the minimum criteria of the protocol format and sample forms. Operational and performance qualification documents should have a clear definition of the minimum criteria of the protocol format, sample forms and the objective and methodology for the applicable testing, inspection procedures and analytical tests. General acceptance criteria that contain the minimum requirements for defining predetermined specifications for the study in question should be part of the standard. Revalidation requirements are required for the type of equipment, system or process to be qualified. A points-to-consider section should anticipate possible variables affecting the qualification study. Sample forms for installation, operational and performance qualification requirements should also be included. Standard documents should contain references used in their development.

The members of the workgroup decided that specific standards need to be developed in the following areas.

- Protocol preparation guidelines for installation, operational and performance qualifications need to be developed.
- Summary report guidelines are needed.
- Master project plan guidelines need to be developed.
- Validation requirements used in the development of purchase or construction specifications are needed.
- Qualification standards are needed for bottled gases, filtered air, potable water, deionized water, water for injection, chilled water, plant steam, clean steam, drainage, kill systems (decontamination), containment systems, classified air systems, heat vacuum air conditioning systems enviromental chambers, environmental monitoring systems, clean in place systems, steam in place systems, automated systems, process vessels, dry heat ovens, sterile filters, autoclaves, ultrafilters, bioreactors, microfiltration processes, incubators, programmable logic systems and chromatography equipment.
- Software functional description guidelines requirements need to be developed.
- Hardware specification guidelines requirements are needed.
- Requirements are needed for software development and qualification.
- Automated system guidelines are needed.
- Cleaning validation requirement guidelines are needed.
- Standards are needed for process qualifications.

## 3. Environment

John Burckle, Senior Research Engineer for the Risk Reduction Engineering Laboratory, Toxic Control Branch of the Enviromental Protection Agency (EPA) chaired the workgroup on industrial biosafety/enviromental aspects of large-scale use of genetically modified organisms and the release of such organisms into the environment. This workgroup concentrated on recently conducted research identified to support the EPA efforts in regulating manufacturing using genetically modified organisms and deliberate enviromental release of these organisms. This research helps to define the areas of standards and provide sufficient data so that standards can be considered. The chair and members identified the following areas as having equally high priority because of their impact on regulatory decision making and because they involve the release of genetically modified organisms to the environment.

- Standards are needed for the methodology of assessing worker exposure to microorganisms in large-scale fermentation facilities. Recent investigations have shown that bioaerosol sampling technology to measure viable and nonviable organisms is inadequate. Basic issues such as which parameters to measure and the most appropriate methods need to be resolved.
- Standards to determine the performance of kill tanks in large-scale facilities need to be resolved because of difficulties in sampling such facilities.
- Guidelines are needed for the detection of genetically engineered microorganisms during field trials. There is a wide variety of test methods that can be used to detect genetically modified organisms. These methods need to be standardized to help facilitate enforcement of current legislation and the development of test guidelines by federal agencies, such as the EPA.
- There is an urgent need to standardize microcosm design and testing protocols for studying the survival and competition of genetically-modified organisms. This will allow the reliable testing of the survival of genetically-modified organisms and their effect on endogenous organisms before they are released into the environment.
- Methods need to be developed to determine the extent of bioremediation by microorganisms. These methods should address preparation, sampling, sample transport, sample preservation, soil moisture, analysis and statistical design including control groups.

## 4. Characterization and Preservation of Living Cells and Viruses

Larry Bockcstahler, Senior Research Biophysictst for the Molecular Biology Branch of the Food and Drug Administration chaired the workgroup on characterization and preservation of living cells and viruses. This workgroup was concerned with standards to characterize the large number of cells and viruses to ensure identity and detect genetic changes during propagation. Voluntary standards in the areas of characterization and preservation assist industrial and other biotechnological laboratories in sustaining high quality control of their products. This workshop reviewed the current standards, protocols and guidance documents for the characterization and preservation of cells and viruses and identified the following specific areas for standards needs in characterization.

- Guides are needed for the cell line identification of mammalian cells based on results of a number of diagnostic tests. This area was given the highest priority. A general guide for polymerase chain reaction for the verification of inserted genes in transgenic animals is also needed.
- In the area of viruses, a specific guide for bacteriophage ΦX-174 along with a draft standard for a new barrier leak testing method should be developed. General guidelines are needed for detecting enveloped RNA, nonenveloped RNA, enveloped DNA, and nonenveloped DNA virus categories. Guidelines for adventitious virus testing and co-cultivation are necessary to help meet Food and Drug Administration requirements and points-to-consider documents. General guidelines for viruses used in gene therapy such as retroviruses and adenoviruses should be developed.
- In the area of bacteria, general standards are needed for “wild-type” industrial isolates used in genetic engineering, including the 10 most commonly used “wild-type” strains and for genetically-engineered bacteria used by the biotechnology industry. In addition, guidelines are needed for sterility testing of finished products of the biotechnology industry.
- For plasmids, guidelines for data interpretation for restriction enzyme mapping of the DNA and determination of copy number to help meet FDA requirements were recommended.
- General guidelines are needed for the identification of fungi used as expression systems in biotechnology.
- A data base is needed for keeping track of the existing standards and to establish better communication between ASTM committee E48 and federal government agencies.

Preservation of living cells and viruses is key to the consistency and quality of biotechnology products and processes. The following specific needs were determined for the preservation section.

- In the area of cell banking, standards are needed to assist users in validating the characterization and preservation processes to satisfy FDA requirements for cell banking. This area was given the highest priority in the preservation section.
- Equipment for low-temperature storage of mammalian cells and inventory control and data management systems such as barcoding and ampule tracking need to be standardized.
- Standards are needed for the proper packing and transport of frozen cells depending on if the use is immediate use or long term storage.
- The banking of tissues from human and other sources requires standards.
- Standards are necessary for safety issues with liquid nitrogen such as oxygen monitoring and ventilation systems.
- Standards for raw material specifications for cryoprotectants and standardized certificates of analysis from vendors are needed.
- The handling of specific hazardous materials during low temperature storage requires standards.

## 5. Safety and Transport of Biomaterials

Frank Simione, Associate Director for Operations at the American Type Culture Collection, chaired the workgroup on safety and transport of biological materials. Safety of biological materials includes their use, transport and disposal. Biological materials can pose safety considerations from their biological, chemical and radiological properties. Transport of biological materials are covered under existing guidelines and regulations but there is a need to improve and clarify the existing documents. The session chair and members identified these areas where standards are needed:

- A safety action plan that considers specific safety issues for a particular product or process should be developed. This area was given the highest priority by the workgroup.
- A general guide for transport standards that covers current regulations and procedures for packaging and shipping of biological materials should be developed. Specific standards would include standards for package performance testing, a guide for determining the level of hazard of an infectious substance and a guide for responding to infectious substances in an accident or during a spill.
- Standard practices for handling hazardous waste are needed that include on site treatment of infectious waste, handling and segregation of chemical and radioactive waste should be developed. Guides are also needed for the treatment and reduction of chemical waste.

## 6. Bioprocess Separations

Gail Sofer, Director of International Validation Development at Pharmacia chaired the workgroup on bioprocess separations. The workgroup agreed that while standards are available from a number of organizations, it is not clear what standards are available and if there is an overlap of existing standards. The group identified the need by industry to consolidate and cross-reference existing standards into a data base. Better communications need to be established among the organizations currently developing standards. The workgroup discussed general guidelines that should determine when a standard is needed. These guidelines included a demonstrated need for a standard, quantitative standards must have a thorough statistical evaluation, standard analytical methods must exist and be validated and standards should not be misapplied or stretched.

General conclusions reached by members of the group were: (1) that the bioseparations area is not yet mature and standards should reflect this, (2) that each biotechnology process is unique and each process requires validation, and (3) that currently, there is not a high demand for standards, but as the industry matures, needs will increase and standards will have to be reviewed often.

The chair and members of the workgroup identified the following specific areas for standard needs.

- Standards for materials classification, especially plastics, are needed. This area was given a high priority.
- A database of existing and developing standards was also given high priority.
- The existing ASTM standard on ultrafiltration membrane performance needs to be upgraded.
- Guides for good development practices are needed.
- Guides to fermentation process development are needed.
- Filtration standards that include stearn-in-place, pressure and cleaning standards and solution compatibility and leachates need to be developed.

## 7. Agriculture/Biomass Workgroup Session

James Walsh of the Enviromental and Technology Laboratory, Georgia Technology Research Institute chaired the agriculture/biomass workgroup. Agricultural and biomass processes generate large volumes of material that must be treated, recycled or disposed of. There is a great amount of interest in the use of agricultural and biomass materials as a renewable source of energy. Standards are necessary to determine the technical and economic performance of energy producing and conversion systems and to characterize feedstock and byproduct materials. Guidance is needed for the design and operation of these processes.

The chair and members of the workgroup have identified the following specific areas for standards needs.

- Development of proposed standards for evaluation of biomass gasifiers should be completed and the terminology document should include definitions developed by the National Renewable Energy Laboratory (NREL).
- A document on standard properties of biomass that could be used for process development to full scale systems should be developed. The document should include a comparison of data from several sources and a standard basis for evaluation of proposals by the Department of Energy.
- Standard testing methods for analysis of cellulose, hemi-cellulose and lignin based on data from NREL should be developed.
- Methods for the analysis of “bio-crude” or oils produced from biomass conversion processes need to be developed.
- Methods for the measurement of lactic and poly-lactic acid produced from biomass are needed.
- A guide is needed for the methods of analysis of simple sugars and sacchanfication processes products.
- A study should be done to determine the precision and accuracy of data from laboratories using existing standards. This study will determine if modifications are needed to these standards.
- Standards are needed for the characterization of the enzymes used in biomass conversion processes.

## 8. Microbiological Diagnostics

Robert James, Director of Quality Assurance and Regulatory Affairs at Becton-Dickson Microbiology Systems was chair of the workshop on microbiological diagnostics. This workgroup reviewed the following general areas where standards might be considered: (1) reference standards including the need by diagnostic manufactures and users and their development and source, (2) specification standards including the minimum standard for assurance, and achieving the minimum standard, (3) calibration standards for manual and automated delivery systems, (4) stability studies including standardized testing protocols for product stability, standardized testing protocols for establishing storage temperature ranges, and storage and usage temperature standard, (5) standardized training programs on use of microbiological diagnostics. Specific attention was paid to the National Committee for Clinical Laboratory Standards (NCCLS), the FDA guidelines for making premarket submissions, and available international standards such as ISO. In addition, the Center for Disease Control and the National Institutes of Health activities relative to microbial standards were reviewed. The chair and members of the workgroup identified the following specific areas where standards are needed.

- A guideline for the handling and maintenance of microbial reference strains by the user is needed. The guideline should include the proper receipt, propagation and storage of strains. In addition, an internal quality control program should include biochemical analysis, antigenic properties, toxin production, virulence factors, and susceptibility characteristics (authenticity). The guide should address replacement of strains to include shelf-life recommendations based on storage conditions and low passage suggestions to minimize mutagenic issues.
- A manufacturer’s guideline for stability of diagnostic kits and reagents is needed. The guideline should include a definition of types of studies (e.g., accelerated studies and its relation to real time studies), use of real time versus accelerated studies, statistically valid study design (that includes number of lots, sample size, time frames, normal and adverse conditions, and cyclic temperature studies), shipping tests to include the use of temperature monitoring devices, labeling for shelf-life and shipping conditions, and packaging considerations.
- A guideline for utilizing thermal cyclers is needed. The guideline should include manufacturing specifications, test methods for manufacturer validation, identification of critical parameters, (e.g., time, temperature, volume), and suggested quality control for the end user. Points to consider for a guideline on thermal cyclers should include allowable tolerances (depending on the design of individual assays), a comparison of thermal blocks, thermal plates and hot air ovens, and a comparison with state of the art equipment.

